# JAZF1 Inhibits Adipose Tissue Macrophages and Adipose Tissue Inflammation in Diet-Induced Diabetic Mice

**DOI:** 10.1155/2018/4507659

**Published:** 2018-03-22

**Authors:** Fanping Meng, Yao Lin, Min Yang, Minyan Li, Gangyi Yang, Po Hao, Ling Li

**Affiliations:** ^1^The Key Laboratory of Laboratory Medical Diagnostics in the Ministry of Education and Department of Clinical Biochemistry, College of Laboratory Medicine, Chongqing Medical University, Chongqing 400010, China; ^2^Department of Endocrinology, The Second Affiliated Hospital, Chongqing Medical University, Chongqing 400010, China; ^3^Department of Laboratory Medicine, Chongqing Three Gorges Medical College, Chongqing 400020, China

## Abstract

**Background:**

Juxtaposed with another zinc finger gene 1 (JAZF1) affects gluconeogenesis, insulin sensitivity, lipid metabolism, and inflammation, but its exact role in chronic inflammation remains unclear. This study aimed to examine JAZF1 overexpression* in vivo* on adipose tissue macrophages (ATMs).

**Methods:**

Mouse models of high-fat diet- (HFD-) induced insulin resistance were induced using C57BL/6J and JAZF1-overexpressing (JAZF1-OX) mice. The mice were randomized (8–10/group) to C57BL/6J mice fed regular diet (RD) (NC group), C57BL/6J mice fed HFD (HF group), JAZF1-OX mice fed RD (NJ group), and JAZF1-OX mice fed HFD (HJ group). Adipose tissue was harvested 12 weeks later. ATMs were evaluated by flow cytometry. Inflammatory markers were evaluated by ELISA.

**Results:**

JAZF1-OX mice had lower blood lipids, blood glucose, body weight, fat weight, and inflammatory markers compared with HF mice (all *P* < 0.05). JAZF1 overexpression decreased ATM number and secretion of proinflammatory cytokines. JAZF1 overexpression decreased total CD4+ T cells, active T cells, and memory T cells and increased Treg cells. JAZF1 overexpression downregulated IFN-*γ* and IL-17 levels and upregulated IL-4 levels. JAZF1 overexpression decreased MHCII, CD40, and CD86 in total ATM, CD11c+ ATM, and CD206+ ATM.

**Conclusions:**

JAZF1 limits adipose tissue inflammation by limiting macrophage populations and restricting their antigen presentation function.

## 1. Introduction

Type 2 diabetes mellitus (T2DM) is a chronic endocrine disorder characterized by hyperglycemia resulting from insulin resistance and deficiency [1]. In patients with T2DM, the chronic hyperglycemic state can lead to multiorgan damage, eventually resulting in renal, neurologic, and cardiovascular complications, among others [1]. The 2014 worldwide prevalence of T2DM was 9% in men and 7.9% in women [2]. In 2010, in China, the prevalence of T2DM was 12.4% in men and 11% in women, and the prevalence of prediabetes was 50.1% [3]. Obesity, hypertension, dyslipidemia, a family history of diabetes, and lifestyle factors are risk factors of T2DM [1, 4]. Over the last few decades, scientific evidence highlighted the complex metabolic and immune pathways that underlie the pathogenesis, progression, and clinical course of T2DM [5].

Recent studies demonstrated that inflammation plays a pivotal role in the occurrence and development of insulin resistance and T2DM [6, 7]. Proinflammatory cytokines (particularly interleukin-6 (IL-6) and tumor necrosis factor-alpha (TNF-*α*)) are chronically increased in diabetic patients and contribute to T2DM development, progression, and complications [3].

Juxtaposed with another zinc finger gene 1 (JAZF1) is an inhibitor of the nuclear receptor subfamily 2, group C, member 2 signaling pathway and has been shown to be involved in gluconeogenesis, insulin sensitivity, lipid metabolism, and inflammation [8]. JAZF1 plays an important role in glucose homeostasis and its overexpression enhances glucose tolerance and insulin sensitivity [8]. The exact mechanisms are poorly understood, but a recent study showed that JAZF1 prevents lipogenesis and systemic inflammatory diseases in transgenic mice [9].

Obesity induces a state of chronic low-grade inflammation, mediated in part by macrophages and other immune cells that populate the adipose tissue, contributing to *β*-cell dysfunction and insulin resistance [10]. The numbers of adipose tissue macrophages (ATMs) increase with body mass and they are the primary cell type regulating inflammation in the context of insulin resistance [11]. The ATMs regulate the secretion of IL-1*β* and IL-18, which play important roles in the development of obesity-related insulin resistance [12]. Macrophage recruitment factors also contribute to ATM infiltration and further development of insulin resistance [13]. Dendritic cells also play important roles (mainly antigen presentation) in the regulation of adipose tissue inflammation [14].

Insulin resistance is associated with immune system dysfunction and chronic low-grade inflammation, but the exact role of JAZF1 in T2DM is still poorly understood. Therefore, this study aimed to determine how JAZF1 affects chronic low-grade inflammation, ATMs, and antigen presentation in a mouse model of diet-induced insulin resistance.

## 2. Materials and Methods

### 2.1. Animals

The mouse model of diet-induced insulin resistance was used to investigate the role of JAZF1* in vivo*. All animal procedures were approved by the ethical committee of Chongqing Medical University. C57BL/6J wild-type mice and JAZF1-OX mice (4 weeks of age; 10–18 g) were used, which is a CMV-promoter-based model [8, 15, 16]. All mice were of the C57BL/6J background. The mice were kept at 18–25°C and humidity of 40–60% under a 12-hour light-dark cycle. The C57BL/6J wild-type mice were obtained from Chongqing Medical University and the JAZF1-OX mice from the West China Center of Medical Sciences, Sichuan University. The mice were placed together in the same cages and acclimatized for 1 week before any experiment.

All mice had free access to water and food throughout the course of the experiment. During the first week, all mice were fed a regular diet (RD). One week later, the mice were randomly divided into four groups of 8–10 mice and fed either a RD or a high-fat diet (HFD): RD-fed wild-type mice as normal control (NC group), HFD-fed wild-type mice (HF group), RD-fed JAZF1-OX mice (NJ group), and HFD-fed JAZF1-OX mice (HJ group) (Supplementary Tables 1 and 2).

After 12 weeks, the mice were sacrificed and weighed. Blood was collected from the left ventricle. Liver, muscle, and heart were obtained. The adipose tissue was harvested from the testicles or womb, kidney, and the abdomen.

### 2.2. Insulin Tolerance Test (ITT) and Glucose Tolerance Test (GTT)

For ITT, after 8 h of fasting, the mice were weighed. Fasting blood glucose levels were measured. Intraperitoneal ITT (insulin load of 0.75 U/kg body weight, Novolin R) was performed. Blood glucose levels were measured at 15, 30, 45, and 60 min from the tail vein.

For GTT, right jugular vein catheterization was conducted 3 days before GTT. GTT was performed when the body weight of the mice recovered to 90% of their preoperative weight. Mice were fasted for 8 h and weighed. Fasting blood samples were collected and fasting blood glucose levels were measured. Intraperitoneal GTT (glucose load of 1.5 g/kg body weight) was performed and blood glucose levels were measured at 15, 30, 60, and 120 min. Blood samples were collected. Plasma was separated by centrifugation and stored at −80°C for plasma insulin (Plns) determination. These tests were carried out in the waking state.

### 2.3. Metabolic Parameters

Blood glucose and lipid metabolism indexes were measured in all mice. Serum total cholesterol (TC), triglycerides (TG), and glucose were measured using a Cobas E600 Automatic Analyzer (Roche Diagnostics, Basel, Switzerland).

### 2.4. Adipose Tissue Isolation

Total adipose tissue was cut using ophthalmic scissors and placed in PBS with 0.25% pancreatin. The mixture was incubated under constant agitation in a 37°C water bath for 1 h. RPMI 1640 culture medium with 2% BSA (Sigma, St. Louis, MO, USA) was added. The mixture was passed through a 40 *μ*m nylon cell strainer (BD Biosciences, Franklin Lake, NJ, USA). Hemolysin was added to remove the erythrocytes. The samples were prepared into single-cell suspensions for flow cytometry.

To monitor the recruitment of macrophages into adipose tissue after 12 weeks of HFD and to look for potential mechanisms of glucose metabolism abnormalities in obese JAZF1-OX mice, the total numbers of ATM, CD206+ ATM, and CD11c+ ATM were compared among the four groups. TNF-*α* and IL-1*β* were measured to detect ATM activity.

### 2.5. Flow Cytometry

The serum inflammation markers were measured according to the manufacturer's instructions using the BD CBA Mouse Th1/Th2 Cytokine Kit (BD Biosciences, Franklin Lake, NJ, USA). The ATM and T cells (1 × 10^6^ cells) were labeled with fluorochrome-conjugated monoclonal antibodies: anti-mouse CD3, CD4, CD11b, CD11c, CD206, F4/80, CD25, CD44, CD69, CD152, FOXP3, MHCII, CD40, CD86, IL-6, IL-1*β*, IL-4, IL-13, IL-17, IL-10, TNF-*α*, and IFN-*γ* (BD Biosciences, Franklin Lake, NJ, USA). For intracellular staining, the cells were activated with PMA/ionomycin (BD Biosciences, Franklin Lake, NJ, USA), according to the manufacturer's instructions. Cells were analyzed with the FACSCalibur Flow Cytometer (BD Biosciences, Franklin Lake, NJ, USA) and the FCAP Array software. Flow cytometry analyses were performed according to standard procedures and according to the manufacturer's instructions.

### 2.6. Statistical Analysis

Data were presented as mean ± SEM and analyzed using one-way ANOVA with the Bonferroni post hoc test. Relationships between pairs of variables were assessed using the Pearson correlation test. Statistical significance was assumed at *P* < 0.05. Statistical analyses were performed using SPSS 18.0 (IBM, Armonk, NY, USA).

## 3. Results

### 3.1. JAZF1-OX Mice Have Lower Blood Lipids, Blood Glucose, Body Weight, and Fat Weight

The body weight, fat weight, blood glucose, TC, and TG were measured to examine the effects of JAZF1 on metabolic parameters. As shown in Table 1, the body weight, epididymal fat weight, blood glucose, TC, and TG were higher in the HF group compared with the NC group (all *P* < 0.05). All these parameters were lower in the HJ group compared with the HF group (all *P* < 0.05).

#### 3.1.1. Insulin Tolerance Test (ITT) and Glucose Tolerance Test (GTT)

**Figure 1:**
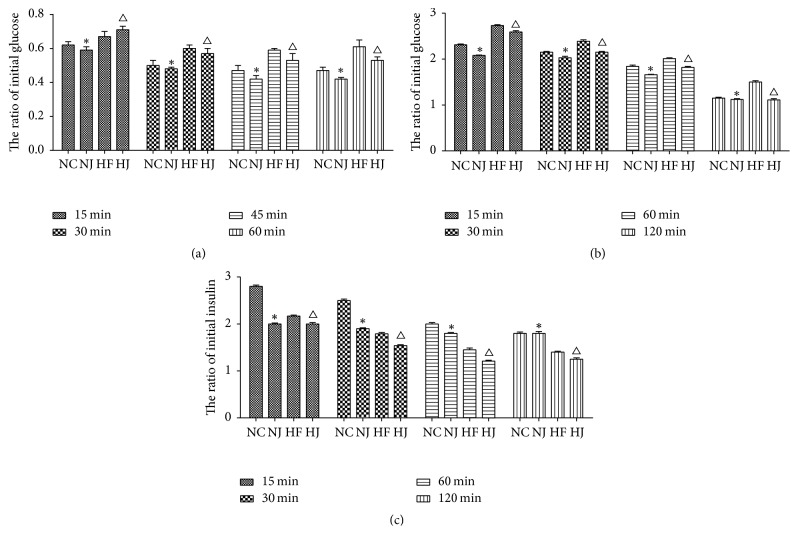
*Blood glucose and insulin levels during ITT and GTT*. (a) The ratio of the initial blood glucose levels during ITT. (b) Time course of the blood glucose levels during GTT. (c) Time course of the blood insulin levels during GTT. ^*∗*^*P* < 0.05 versus the NC group. ^Δ^*P* < 0.05 versus the HF group. Values are presented as mean ± SEM (*n* = 8–10/group).

ITT and GTT were performed to examine the effect of JAZF1 on glucose metabolism. For ITT, after intraperitoneal injection of insulin, the mice showed lower blood glucose levels compared with baseline (0 min) (*P* < 0.05). The blood glucose levels in the NJ group at 30 and 60 min were significantly lower than those in the NC group (*P* < 0.05). After injection of insulin, mice in the HJ group showed significantly decreased blood glucose levels at all time points (15, 30, 45, and 60 min) compared to the HF group (*P* < 0.05 or *P* < 0.01) (Figure 1(a)).

For the GTT analysis, after intraperitoneal injection of glucose, blood glucose levels were significantly increased in all groups compared with baseline (0 min) (*P* < 0.05). The blood glucose levels of the NJ group were significantly lower than those in the mice of the NC group at 15 min (*P* < 0.05), but the insulin levels at 15, 30, and 45 min of the NJ group were significantly lower than the insulin levels in the NC group (*P* < 0.01). The mice in the HJ group showed significantly lower blood glucose and insulin levels than those in the HF group at 15 and 120 min (*P* < 0.01) (Figures 1(b)-1(c)).

### 3.2. JAZF1-OX Mice Have Lower Inflammatory Markers

**Figure 2:**
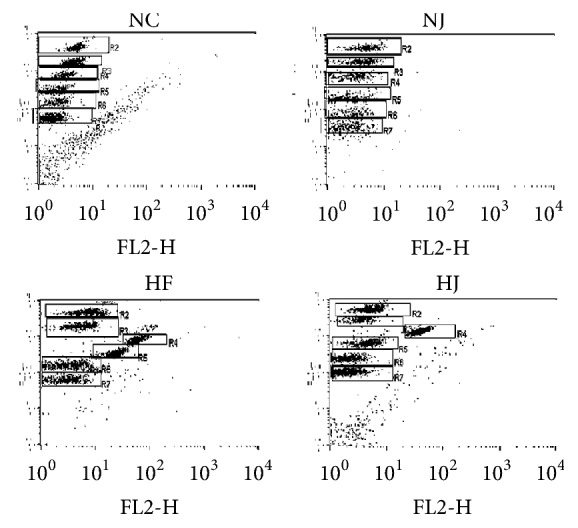
*Plasma inflammation factors levels in mice*. Flow cytometry representation of IL-4 (gate R3), IL-6 (gate R4), IL-10 (gate R5), TNF-*α* (gate R6), and IFN-*γ* (gate R7) in each group.

The expression levels of IL-4, IL-6, IL-10, TNF-*α*, and IFN-*γ* were measured to examine the effects of JAZF1 on inflammation. The levels of IL-4, IL-6, IL-10, TNF-*α*, and IFN-*γ* were higher in the HF group compared with the NC group (all *P* < 0.05), while all these parameters were lower in the HJ group compared with the HF group (all *P* < 0.05) (Table 2 and Figure 2).

### 3.3. JAZF1 Decreases ATM Numbers and Their Cytokine Secretion

**Figure 3:**
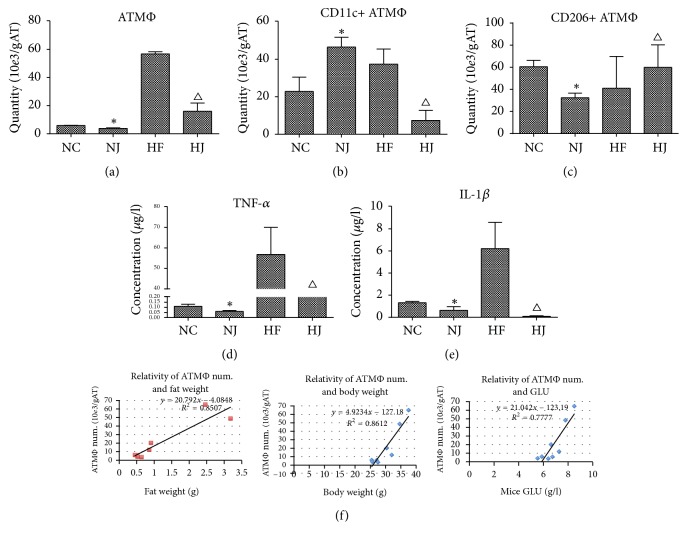
*Adipose tissue macrophages (ATM) in mice (from epididymal fat)*. (a) Total ATM, (b) CD11c+ ATM and (c) CD206+ ATM numbers, and (d) TNF-*α* and (e) IL-1*β* levels in ATM. (f) Positive correlation between ATM and fat mass, body weight, and fasting blood glucose levels in HFD mice. Studies were performed at 16 weeks of age. Values are presented as mean ± SEM (*n* = 8–10/group). ^*∗*^*P* < 0.05 versus the NC group; ^Δ^*P* < 0.05 versus the HF group.

The numbers of total ATM and CD11c+ ATM and the secretion of TNF-*α* and IL-1*β* were measured to assess the effect of JAZF1 on immune cells. Total ATM numbers, CD11c+ ATM numbers, TNF-*α*, and IL-1*β* in the HF group were higher than those in the NC group (all *P* < 0.05), while these markers were lower in the HJ group than in the HF group (all *P* < 0.05). For CD206+ ATM, the levels were lower in the HF group compared with the NC and HJ groups (all *P* < 0.05), suggesting that JAZF1 can decrease CD11c+ ATMs but enhances CD206+ ATMs. The parameters in the NJ and NC groups were not significantly different (*P* > 0.05) (Figures 3(a)–3(e), Supplementary Figure 1).

The correlations between the ATM numbers and fat mass, body weight, and glucose were examined.  Figure 3(f) shows that, in HFD mice, the total ATM numbers were positively correlated with fat mass (*r*^2^ = 0.85), body weight (*r*^2^ = 0.86), and fasting blood glucose (*r*^2^ = 0.77).

### 3.4. JAZF1 Lowers Total CD4+ T Cells, CD69+ Active T Cells, and CD44+ Memory T Cells and Enhances CD25+ FOXP3+ Treg Cells

**Figure 4:**
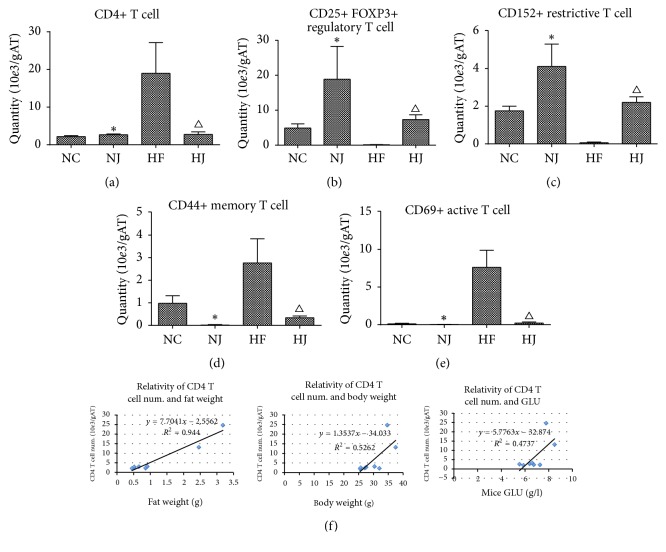
*T cells in mice (from epididymal fat)*. (a) Total CD4+ AT T cell numbers. (b) Total CD25+ FOXP3+ regulatory T cell numbers. (c) Total CD152 regulatory T cell numbers. (d) Total CD44+ memory T cell numbers. (e) CD69+ active T cell numbers. (f) Positive correlation between the CD4+ AT T cell number and fat mass, body weight, and fasting blood glucose levels in HFD mice. Values are presented as mean ± SEM (*n* = 8–10/group). ^*∗*^*P* < 0.05 versus the NC group; ^Δ^*P* < 0.05 versus the HF group.

As JAZF1-OX ATMs produce less CD4+ T cell-activating cytokines (Table 2), these cells were evaluated. The total CD4+ T cell number, CD69+ activated T cell number, and CD44+ memory T cells were higher in the HF group than in the NC group (*P* < 0.05) and lower in the HJ group compared with the HF group (*P* < 0.05). With regard to the CD25+ FOXP3+ Treg cell numbers and total T cell numbers, the HF group had lower counts than the NC and HJ groups (*P* < 0.05). The parameters in the NJ and NC groups were not significantly different (*P* > 0.05) (Figures 4(a)–4(e), Supplementary Figure 2).

There were correlations between CD4+ AT T cell numbers, fat mass, body weight, and fasting glucose. The total CD4+ AT T cell counts were positively correlated with fat mass (*r*^2^ = 0.94), body weight (*r*^2^ = 0.52), and fasting blood glucose levels (*r*^2^ = 0.47) (Figure 4(f)).

### 3.5. JAZF1 Downregulates IFN-*γ* and IL-17 Levels and Upregulates IL-4 Levels

**Figure 5:**
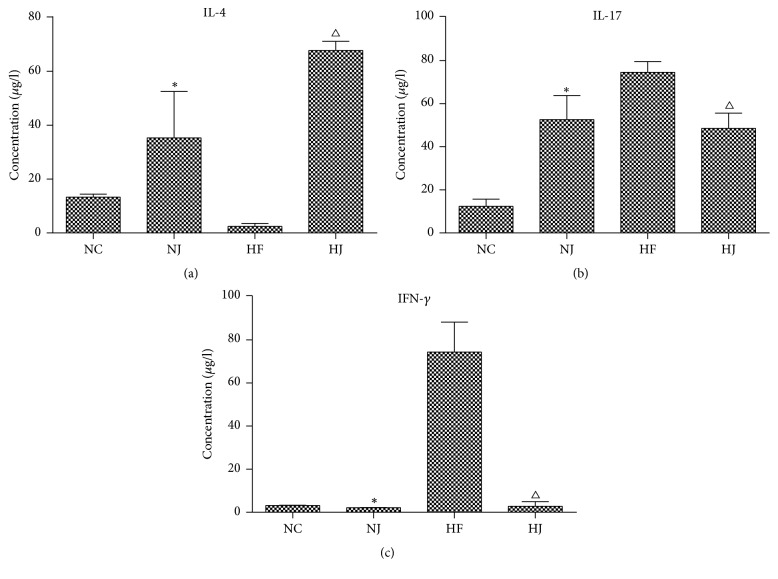
*Inflammatory markers of CD4 T cells in mice (from epididymal fat)*. Levels of (a) IL-4, (b) IL-17, and (c) IFN-*γ* from epididymal fat cell suspension. Values are presented as mean ± SEM (*n* = 8–10/group). ^*∗*^*P* < 0.05 versus the NC group; ^Δ^*P* < 0.05 versus the HF group.

IFN-*γ* and IL-17 were measured to examine the effect of JAZF1 on the markers of inflammation. CD4+ T cells in the HF group secreted more IFN-*γ* and IL-17 than those in the NC and HJ groups. The HF group had lower levels of IL-4 than the NC and HJ groups, while the NC and NJ groups showed no significant differences in these parameters (*P* > 0.05) (Figure 5, Supplementary Figure 3).

### 3.6. JAZF1 Lowers MHCII, CD40, and CD86 in Total ATM, CD11c+ ATM, and CD206+ ATM

**Figure 6:**
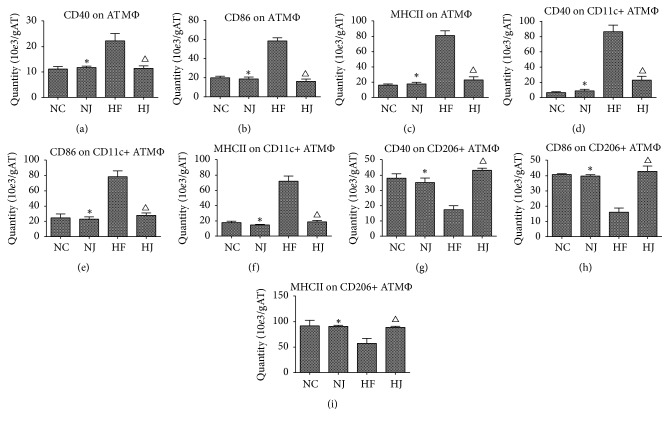
*MHCII among ATM in mice (from epididymal fat)*. (a) The total number of CD40 on ATM. (b) The total number of CD86 on ATM. (c) The total number of MHCII on ATM. (d) The total number of CD40 on CD11c+ ATM. (e) The total number of CD86 on CD11c+ ATM. (f) The total number of CD40 on CD11c+ ATM. (g) The total number of CD40 on CD206+ ATM. (h) The total number of CD86 on CD206+ ATM. (i) The total number of MHCII on CD206+ ATM. Values are presented as mean ± SEM (*n* = 8–10/group). ^*∗*^*P* < 0.05 versus the NC group; ^Δ^*P* < 0.05 versus the HF group.

MHCII and costimulatory molecules (CD86 and CD40) were measured in order to examine the effect of JAZF1 on T cell activation. MHCII and costimulatory molecules (CD86 and CD40) are required for ATM-mediated CD4+ T cell activation; they were significantly decreased in JAZF1-OX ATM and CD11c+ ATM but were increased in CD206+ ATM (*P* < 0.05) (Figures 6(a)–6(i)). MHCII, CD40, and CD86 in total ATM and CD11c+ ATM in the HF group were higher than those in the NC group (*P* < 0.05) but lower than those in the HJ group (*P* < 0.05). CD206+ ATMs in the HF group were lower than those in the NC and HJ groups. There were no significant differences between the NJ and NC groups (*P* > 0.05) (Figures 6(a)–6(i), Supplementary Figure 4).

## 4. Discussion

JAZF1 affects gluconeogenesis, insulin sensitivity, lipid metabolism, and inflammation, but its exact role in chronic inflammation remains unclear. Therefore, this study examined whether JAZF1 overexpression* in vivo* can decrease ATMs. JAZF1 limits adipose tissue inflammation by limiting macrophage populations and restricting their antigen presentation function. This would play a role in the development and progression of T2DM.

In recent years, studies have highlighted many aspects of the complex interactions among genetic, environmental, metabolic, and immune factors that contribute to the development and progression of T2DM. We now know that diseases that fall into the metabolic syndrome spectrum, such as T2DM and obesity, are associated with dysfunctional immunity and low low-grade inflammation, as shown by increased levels of proinflammatory cytokines such as TNF-*α*, IL-6, and chemotactic factors (e.g., CCL5 and CCL8) [6, 7, 12]. Inhibition of key proinflammatory cytokines has been shown,* in vivo*, to protect rodents from insulin resistance and T2DM [17]. This proinflammatory response has been further associated with an imbalanced T cell subtype differentiation, particularly decreased CD4+ subtypes traditionally associated with chronic inflammation [18].

The present study showed that, compared with normal control mice fed a RD, mice fed a HFD (HF group) had higher levels of IFN-*γ* and IL-17 expressed by CD4+ T cells, while IL-4 produced from Treg cells was reduced. In addition, the total numbers of CD4+ T cells and of activated and memory T cell were higher in mice fed a HFD than in those fed a RD, which suggests changes in CD4+ T cell subtype differentiation taking place in adipose tissue. Feeding high fat diets to mice gave rise to low-grade inflammation in the adipose tissue, leading in turn to the recruitment of immune cells and production of proinflammatory cytokines leading to insulin resistance [19–21]. Controlling low-grade inflammation in adipocytes should be considered as a targeted approach to limit the pathogenesis of metabolic diseases.

Studies in rats show that increased plasma FFA can activate muscle NF-*κ*B signaling [22], which can lead to higher levels of proinflammatory cytokines such as TNF-*α*, IL-1*β*, IL-6, and MCP-1 [23]. This increase in plasma MCP-1 can promote monocyte migration from the blood to the adipose tissue. When macrophages settle in the adipose tissue, they differentiate into inflammatory macrophages, which can release a great amount of inflammatory factors [24, 25]. ATF2 has a similar effect, and total and phosphorylated ATF2 are highly expressed in infiltrated macrophages [26]. Macrophages can be classified into M1 and M2. M1 macrophages are activated by the classical pathway and promote inflammation, while M2 macrophages regulate inflammation and are activated by alternative pathways. A previous study showed that the macrophages' subtype differentiation was changed in the adipose tissue, where M2 macrophages tended to transform into M1 macrophages [27]. Proinflammatory ATMs, which express CD11c+ (a classical marker for activated M1), are generally increased, hereby leading to inflammation and insulin resistance [28–31], resulting in a reduction of the relative number of CD206+ macrophages (alternatively activated or M2 macrophages), which are predominantly anti-inflammatory [32, 33]. In the present study, the HF group had higher levels of total ATM counts, CD11c+ ATM, and TNF-*α* and IL-1*β* compared to mice fed a RD. The CD206+ ATM parameters were lower in the HF group than in the NC and HJ groups, supporting the hypothesis that the macrophage activation pathways had switched to favor proinflammatory macrophages. In JAZF1-OX mice fed a HFD, CD4+ T cell and macrophage counts, secretion, and subtype differentiation were at the opposite of the results observed in wild-type mice fed the same diet. Nevertheless, a recent study suggests that IL-6 regulates M2 differentiation in ATM in obesity [34]. Additional studies are necessary to elucidate the exact mechanisms involved in M1/M2 differentiation.

Previous studies showed that JAZF1 is closely associated with insulin secretion and sensitivity in T2DM, as well as with *β*-cell function [35, 36]. Previous studies by our group found that JAZF1 could promote lipid accumulation of 3T3-L1 cells and upregulate the expression of GLUT1 in hepatocytes [9, 15, 37]. JAZF1 transgenic mice showed that overexpression of JAZF1 can downregulate PEPCK and G6Pase levels (which are key enzymes in glucose metabolism) and increase the insulin receptor substrate-1 (IRS-1) level and phosphorylation [9, 15, 37], suggesting that JAZF1 may increase the rate of basal glucose transport in hepatocytes. Our previous work also shows that upregulation of JAZF1 in hepatocytes can*, in vivo*, inhibit the expression of proinflammatory cytokines such as TNF-*α*, MCP-1, and IL-8 via the action of palmitic acid [9, 15, 37]. In the meantime, JNK, p38 MAPK, and NF-*κ*B may also take part in this process. The present study supports the accumulating evidence of the association between JAZF1 and inflammation. Moreover, JAZF1 appears to be a key player in metabolic gene expression and T2DM development and, as such, its role needs to be further studied to clarify its implications in inflammation [38, 39].

A study showed that the adipose tissue in CD40-deficient animals has elevated cytokine levels and inflammatory cell infiltrates, particularly of macrophages and T cells [40], suggesting an important role of TH1 cells in regulating inflammation and insulin resistance in obesity. CD4+ and CD8+ T cells populate the human adipose tissue and the Th1:Th2 balance is highly associated with systemic inflammation and insulin resistance; in addition, MHCII and costimulators CD86 and CD40 play essential roles in obesity-induced adipose tissue inflammation [41].

Taken together, these findings suggest that the adaptive immune system could be a potential mediator between obesity and insulin resistance or inflammation [42–45]. MHCII and costimulatory molecules (CD86 and CD40) required for ATM-mediated CD4+ T cell activation were evaluated and the results showed that they were significantly increased in JAZF1-OX ATM. In addition, MHCII, CD40, and CD86 in total ATM and CD11c+ ATM counts were higher in HFD-fed mice than in control mice and those in HFD-fed JAZF1-OX mice. Concerning CD206+ ATM, the parameters in the HF group were lower than in the NC and HJ groups. Nevertheless, much work is still necessary to examine the exact interactions between JAZF1 and specific immune cells. A previous study showed that JAZF1 transgenic mice have a natural resistance to HFD-induced obesity [39]. In addition, the same study showed that JAZF1 plays an important role in lipid homeostasis under HFD conditions [39]. Since overweight and blood lipids are involved in inflammation, JAZF1 overexpression could decrease the inflammatory state under HFD. Indeed, in the present study, no differences were observed between the NC and HJ groups, indicating that JAZF1 played some role in protecting the mice against the deleterious effects of HFD. The main differences were observed between the HC and HJ groups. Finally, the present study was not designed to identify the exact mechanisms involved in the observed effects of JAZF1 on immune cells. A previous study showed that the JNK pathway might be involved [9], providing a starting point for future experiments. Additional studies are necessary to address these issues.

## 5. Conclusions

HFD-fed JAZF1-OX mice showed lower levels of ATM, especially the CD11c+ ATM and the proinflammatory cytokines they secrete, compared with wild-type mice fed the same diet. Similar observations were made for CD4+ T cells and their proinflammatory cytokines, MHCII, CD40, and CD86. Taken together, these results suggest that JAZF1 can control ATMs by downregulating their antigen presentation function and lowering adipose tissue inflammation.

## Figures and Tables

**Table 1:** Metabolic characteristics of the animals.

Metabolic parameter	NC	NJ	HF	HJ
Body weight (g)	26.31 ± 0.81	26.59 ± 0.91	36.09 ± 1.43^*∗*^	31.21 ± 0.81^Δ^
Epididymal fat weight (g)	0.48 ± 0.04	0.58 ± 0.08	2.82 ± 0.51^*∗*^	0.89 ± 0.04^Δ^
Glucose (mM)	6.30 ± 0.43	5.94 ± 0.43	8.15 ± 0.36^*∗*^	6.96 ± 0.32^Δ^
TC (mM)	2.56 ± 0.09	2.17 ± 0.09	3.82 ± 0.08^*∗*^	3.07 ± 0.07^Δ^
TG (mM)	0.58 ± 0.04	0.51 ± 0.04	0.83 ± 0.03^*∗*^	0.73 ± 0.05^Δ^

Values are presented as mean ± SEM (*n* = 6 per group). TC: total cholesterol; TG: triglycerides; NC: wild-type mice fed a regular diet; HF: wild-type mice fed a high-fat diet; NJ: JAZF1-OX mice fed a regular diet; HJ: JAZF1-OX mice fed a high-fat diet. ^*∗*^*P* < 0.05 versus the NC group; ^Δ^*P* < 0.05 versus the HF group.

**Table 2:** Plasma inflammatory factors.

	IL-4	IL-6	IL-10	TNF-*α*	IFN-*γ*
NC	4.44 ± 0.22	2.84 ± 0.64	2.63 ± 0.16	2.64 ± 0.01	2.55 ± 0.01
NJ	4.46 ± 0.07	2.92 ± 0.33	2.30 ± 0.23	2.39 ± 0.32	2.10 ± 0.46
HF	6.16 ± 0.36^*∗*^	73.49 ± 23.12^*∗*^	15.79 ± 11.65^*∗*^	4.23 ± 0.05^*∗*^	3.40 ± 0.11^*∗*^
HJ	3.67 ± 0.20^Δ^	23.79 ± 19.00^Δ^	5.19 ± 1.12^Δ^	2.39 ± 0.05^Δ^	2.32 ± 0.02^Δ^

Results are presented as mean ± SEM of three experiments. *n* = 8–10/group. IL: interleukin; TNF-*α*: tumor necrosis factor-*α*; IFN-*γ*: interferon-*γ*; NC: wild-type mice fed a regular diet; HF: wild-type mice fed a high-fat diet; NJ: JAZF1-OX mice fed a regular diet; HJ: JAZF1-OX mice fed a high-fat diet. ^*∗*^*P* < 0.05 versus the NC group; ^Δ^*P* versus the NJ group.

## Supplementary Materials

Supplementary Materials Supplementary Table 1: composition of the regular diet (RD) (per 1000 g). Supplementary Table 2: comparison of the weight and energy compositions between the regular diet (RD) and high-fat diet (HFD). Supplementary Figure 1: flow cytometry representation of gated (A) ATM (CD11b+F4/80+), (B) IL-1*β*, and (C) TNF-*α* intracellular staining in ATM and CD11c+ and CD206+ ATM. Supplementary Figure 2: flow cytometry representation of gated AT (A) CD4 T cells (CD3+ CD4+), (B) Treg T cells (CD25+ FOXP3+), (C) active T cells (CD4+ CD69+), (D) restrained T cells (CD4+ CD152+), and (E) memory T cells (CD4+ CD44+). Supplementary Figure 3: flow cytometry representation of gated AT CD4 T cells stained intracellularly with (A) IL-4 and (B) IL-17. Supplementary Figure 4: costimulatory molecule (CD40 and CD86) expression in gated total ATM, MHCII, and costimulatory molecule (CD40, CD80, and CD86) expression in gated CD11c+ ATM, MHCII, and costimulatory molecule (CD40, CD80, and CD86) expression in gated CD206+ ATM (*n* = 8–10/group). (B) Flow cytometry representation of gated MHCII in ATM, CD40 and CD86 in ATM, gated MHCII in CD11c+ ATM, CD40 in CD11c+ ATM, CD86 in CD11c+ ATM, MHCII in CD206+ ATM, CD40 in CD206+ ATM, and CD86 in CD206+ ATM (*n* = 8–10/group).
