# Supplementary material for: JAZF1 Inhibits Adipose Tissue Macrophages and Adipose Tissue Inflammation in Diet-Induced Diabetic Mice
Source: Biomed Res Int. 2018 Mar 22;2018:4507659. doi: 10.1155/2018/4507659 (PMC5885486; doi:10.1155/2018/4507659)
Supplement: Supplementary Materials — Supplementary Table 1: composition of the regular diet (RD) (per 1000 g). Supplementary Table 2: comparison of the weight and energy compositions between the regular diet (RD) and high-fat diet (HFD). Supplementary Figure 1: flow cytometry representation of gated (A) ATM (CD11b+F4/80+), (B) IL-1β, and (C) TNF-α intracellular staining in ATM and CD11c+ and CD206+ ATM. Supplementary Figure 2: flow cytometry representation of gated AT (A) CD4 T cells (CD3+ CD4+), (B) Treg T cells (CD25+ FOXP3+), (C) active T cells (CD4+ CD69+), (D) restrained T cells (CD4+ CD152+), and (E) memory T cells (CD4+ CD44+). Supplementary Figure 3: flow cytometry representation of gated AT CD4 T cells stained intracellularly with (A) IL-4 and (B) IL-17. Supplementary Figure 4: costimulatory molecule (CD40 and CD86) expression in gated total ATM, MHCII, and costimulatory molecule (CD40, CD80, and CD86) expression in gated CD11c+ ATM, MHCII, and costimulatory molecule (CD40, CD80, and CD86) expression in gated CD206+ ATM (n = 8–10/group). (B) Flow cytometry representation of gated MHCII in ATM, CD40 and CD86 in ATM, gated MHCII in CD11c+ ATM, CD40 in CD11c+ ATM, CD86 in CD11c+ ATM, MHCII in CD206+ ATM, CD40 in CD206+ ATM, and CD86 in CD206+ ATM (n = 8–10/group). [file 4507659.f1.zip › 4507659.f1/4507659.f5.docx]

Supplementary Table 1. Composition of the regular diet (RD) (per 1000 g)

| Component | Content (g) | Component | Content (g) |
| --- | --- | --- | --- |
| Corn | 330 | Milk powder | 20 |
| Soybean | 150 | Egg | 50 |
| Sesame seed cake | 100 | Yeast | 20 |
| Wheat bran | 150 | Salt | 50 |
| Flour | 150 | Bone meal | 25 |
| Compound vitamins | 0. 3 |  |  |
